# Transforming Human Immunodeficiency Virus Outcomes: Impact of a Rapid Antiretroviral Therapy Model on Linkage to Care and Viral Suppression in Memphis, Tennessee

**DOI:** 10.1093/ofid/ofag506

**Published:** 2026-08-19

**Authors:** April C Pettit, Paridhi Ranadive, Aima A Ahonkhai, Agatha Eke, Damian J Denson, Leslie J Pierce, Latrice C Pichon, Brittany Wilbourn, Carolyn M Audet, Peter F Rebeiro

**Affiliations:** Department of Medicine, Vanderbilt University Medical Center, Nashville, Tennessee, USA; Department of Biostatistics, Vanderbilt University Medical Center, Nashville, Tennessee, USA; Department of Medicine, Massachusetts General Hospital, Boston, Massachusetts, USA; National Center for HIV, Viral Hepatitis, STD, and Tuberculosis Prevention, Division of HIV Prevention, Centers for Disease Control and Prevention, Atlanta, Georgia, USA; National Center for HIV, Viral Hepatitis, STD, and Tuberculosis Prevention, Division of HIV Prevention, Centers for Disease Control and Prevention, Atlanta, Georgia, USA; Department of Medicine, Massachusetts General Hospital, Boston, Massachusetts, USA; Division of Social and Behavioral Sciences, University of Memphis School of Public Health, Memphis, Tennessee, USA; National Center for HIV, Viral Hepatitis, STD, and Tuberculosis Prevention, Division of HIV Prevention, Centers for Disease Control and Prevention, Atlanta, Georgia, USA; Department of Health Policy, Vanderbilt University Medical Center, Nashville, Tennessee, USA; Department of Medicine, Vanderbilt University Medical Center, Nashville, Tennessee, USA

**Keywords:** human immunodeficiency virus, rapid antiretroviral therapy, linkage to care, viral suppression, United States South

## Abstract

**Background:**

In May 2024, a locally tailored, multicomponent rapid antiretroviral therapy intervention—Connextion—was launched in Memphis, Tennessee, a priority jurisdiction of the Ending the HIV Epidemic in the US initiative. This study assessed its impact on time to care linkage and viral suppression among newly diagnosed individuals with human immunodeficiency virus (HIV).

**Methods:**

We included adults newly diagnosed with HIV between January 2022 and May 2025 at participating Connextion sites, who were identified through the Tennessee Enhanced HIV/AIDS Reporting System (eHARS). Adjusted Cox proportional hazards models were used to estimate adjusted hazard ratios (aHRs) for time to linkage to care, defined as the interval from diagnosis to the first recorded CD4^+^ cell count or HIV RNA level and time to viral suppression, defined as the interval from diagnosis to the first HIV RNA level <200 copies/mL.

**Results:**

We included 278 individuals newly diagnosed with HIV; median age was 30 years, 71% were male, 75% were Black non-Hispanic, and 41% identified as men who have sex with men. In the post-implementation period (beginning June 2024), there was a significant increase in the adjusted probability of linkage to care (aHR, 1.76 [95% CI, 1.31–2.36]) and viral suppression (aHR, 4.22 [95% CI, 2.67–6.69]).

**Conclusions:**

After implementation of Connextion, there was a reduction in time to linkage to care and viral suppression. Future plans include expanding the intervention to additional HIV testing and treatment sites in Memphis and broadening eligibility to include people living with HIV who are out of care.

## Teaser

Launched in May 2024, the Connextion rapid antiretroviral therapy intervention in Memphis, Tennessee, significantly reduced time to care linkage and viral suppression among people newly diagnosed with HIV. Results support expanding the intervention to more sites and populations.

Rapid antiretroviral therapy (ART) initiation is an evidence-based intervention in which people newly diagnosed with human immunodeficiency virus (HIV) start treatment as soon as possible after HIV diagnosis [1]. Rapid ART initiation has been shown to improve both individual and population-level HIV outcomes, by reducing morbidity and mortality of people with HIV and decreasing HIV transmission [2]. Thus, it is a vital component of the United States (US) and local Memphis Ending the HIV Epidemic (EHE) Initiatives [3, 4] and is recommended in international [5] and national [6] HIV treatment guidelines.

Like many evidence-based interventions, rapid ART initiation faces barriers to uptake in practice. Our previous work and other studies have reported challenges including insufficient provider capacity, lack of required documentation or laboratory results, lack of provider training, and patient obstacles due to social determinants of health [7–13]. Additionally, specific barriers, their relative contribution to treatment delays, and optimal strategies to overcome them vary by clinical site [14].

One way that implementation science improves the uptake of evidence-based interventions—such as rapid ART initiation—is by ensuring the strategies used to increase uptake are tailored to the local context [15]. Guided by implementation science methods, we previously developed a locally tailored toolkit to support an increase in rapid ART initiation in Memphis, Tennessee [7]. Specifically, we conducted process mapping guided by the Consolidated Framework for Implementation Research to outline the steps from HIV testing to HIV treatment sites in Memphis, identifying barriers leading to delays in care. We then used modified conjoint analyses to prioritize barriers and identify strategies for improving rapid ART implementation, focusing on the potential impact and feasibility of changes.

Notably, this toolkit was implemented in a region of the country that accounts for nearly half of people newly diagnosed with HIV [16], in a state without Medicaid expansion [17], in a metropolitan statistical area (MSA) with high poverty rates (16.5% vs 12.5% below the poverty line in Memphis vs US, respectively) [18], and an MSA ranked one of the highest in the US for HIV incidence [19]. The intervention, locally referred to as Connextion (English) or Connextión (Spanish), consists of several implementation strategies including rapid ART staff trainings, double rapid HIV testing procedures (to prevent delays in confirmatory testing), a “warm hand-off” of people newly diagnosed with HIV from testing to treatment facilities, stigma reduction activities, and the use of pharmaceutical samples when needed [7]. A standard operating procedures (SOP) document that serves as a practical guide for implementing the aforementioned strategies provides intervention details [20].

Connextion was implemented by 5 participating sites that provide HIV testing and/or treatment in Memphis, Tennessee. Here, we use public health surveillance data to identify changes in times to linkage to HIV care and viral suppression 1 year following intervention implementation. We also assess outcomes by site and demographic characteristics to inform future intervention refinements tailoring the intervention for important subgroups.

## METHODS

### Study Population and Setting

We conducted a retrospective cohort study among adults ≥18 years of age newly diagnosed with HIV who were tested and linked to care at 1 of 5 participating Connextion sites in Memphis, Tennessee from January 2022 to May 2025. Participating sites included (1) a community-based organization (CBO), (2) a faith-based federally qualified health center (FQHC), (3) the municipal public health department, (4) an adult university-affiliated HIV clinic, and (5) a pediatric infectious diseases specialty clinic that provides all pediatric HIV care in Memphis, including patients newly diagnosed with HIV ≤21 years of age.

On 31 May 2024, memorandums of understanding (MOUs) were executed for the 5 participating sites, and the post-implementation period began 1 June 2024 and ended 31 May 2025. All sites except the pediatric clinic conduct HIV testing; the CBO testing site was already conducting rapid confirmatory HIV testing prior to the Connextion pre-implementation period, and the health department site added this service in the post-implementation period. All sites except the CBO and health department provide HIV treatment services. At the time of Connextion implementation, 2 of the 3 participating HIV treatment sites (adult and pediatric HIV clinics) had some providers who were already providing ART initiation at the first HIV treatment visit (although not on the day of HIV diagnosis). The third site (an FQHC) added this service in the post-implementation period. While there were differences across sites regarding HIV testing and treatment services, all sites utilized strategies in the SOP with respect to rapid ART staff trainings and identifying a point of contact for a “warm hand-off” of people newly diagnosed with HIV from testing to treatment facilities, ideally on the same day of HIV diagnosis (Table 1). All HIV treatment sites utilize bictegravir, emtricitabine, and tenofovir alafenamide as a first-line rapid ART initiation regimen.

**Table 1.** HIV Services Provided by Participating Connextion Sites in the Pre- and Post-implementation Periods

Site	Pre-implementation Period (Jan 2022–May 2024)	Post-implementation Period (June 2024–May 2025)	Change in Services
CBO	HIV testing (including rapid confirmatory testing) No HIV treatment services	HIV testing (including rapid confirmatory testing) No HIV treatment services	None
FQHC	HIV testing (without rapid confirmatory testing) HIV treatment (without rapid ART initiation)	HIV testing (without rapid confirmatory testing) HIV treatment (including rapid ART initiation using samples when needed)	Added rapid ART initiation using samples when needed
Municipal Health Department	HIV testing (without rapid ART initiation) No HIV treatment services	HIV testing (including rapid confirmatory testing) No HIV treatment services	Added rapid confirmatory testing
Adult HIV Clinic	HIV testing (without rapid confirmatory testing) HIV treatment (including rapid ART initiation using samples when needed)	HIV testing (without rapid confirmatory testing) HIV treatment (including rapid ART initiation using samples when needed)	No change
Pediatric HIV Clinic	No HIV testing services HIV treatment (including rapid ART initiation using samples when needed)	No HIV testing services HIV treatment (including rapid ART initiation using samples when needed)	No change

Abbreviations: ART, antiretroviral therapy; CBO, community-based organization; FQHC, federally qualified health center; HIV, human immunodeficiency virus.

Eligible participants were identified using the Tennessee Enhanced HIV/AIDS Reporting System (eHARS). Participants were followed from the date of HIV diagnosis until the two primary outcomes (linkage to HIV care and viral suppression, defined below), death, or database closure on 31 May 2025. This study was approved by the institutional review boards at Vanderbilt University Medical Center (220932) and the Tennessee Department of Health (2022-0335).

### Study Definitions

There were two primary outcomes of interest: time to linkage to care and time to viral suppression. Time to linkage to care was defined as the time from HIV diagnosis to the first available CD4^+^ count or HIV RNA measurement (viral load) in eHARS. Individuals who had no available laboratory results following HIV diagnosis were followed until death, database closure (31 May 2025), or 12 months of follow-up, whichever came first. All individuals were allowed a maximum of 12 months of follow-up after HIV diagnosis in both the pre- and post-implementation periods so the relative times at risk for each outcome were comparable between periods. Time to viral suppression was defined as the time from HIV diagnosis to the first viral load <200 copies/mL; 18 (6.5%) of individuals had no viral load measured or never had a suppressed viral load, and they were censored at 12 months of follow-up and contributed all their person-time as event free.

We also extracted demographic characteristics including age, sex, race/ethnicity, HIV acquisition risk factor, HIV testing site, HIV linkage site, baseline HIV laboratory values, and Ryan White HIV/AIDS Program (RWHAP) enrollment. Age was defined as age in years at the time of HIV diagnosis. Sex was categorized as male, female, or unknown. Race/ethnicity was categorized as Black, non-Hispanic; White, non-Hispanic; Hispanic; or Other/Unknown. HIV acquisition risk factor was categorized as male-to-male sexual contact (MSM), heterosexual contact, injection drug use, or other/unknown. Baseline laboratory results, including CD4^+^ counts and viral loads, were defined as those laboratory values closest to HIV diagnosis and not more than 90 days following diagnosis. RWHAP enrollment status, which serves as a proxy for socioeconomic status given that eligibility requires a gross household income ≤400% of the federal poverty level in Tennessee, was categorized as enrolled or not enrolled during the first 12 months following HIV diagnosis [7].

### Statistical Analysis

We used frequency and proportion or median and interquartile range (IQR) to describe categorical and continuous variables, respectively. Differences in categorical and continuous variables in the pre- versus post-implementation periods were tested using the Fisher exact and Wilcoxon rank-sum tests, respectively. *P* values were 2-sided and considered statistically significant if <.05.

We used log-rank tests to compare the Kaplan-Meier curves for time until linkage to care and viral suppression between the pre- and post-implementation periods; individuals were censored at 12 months of follow-up post–HIV diagnosis if they did not have a laboratory measurement prior. Multivariable Cox proportional hazards models were used to estimate the adjusted hazard ratios (aHRs) and 95% confidence intervals (CIs) for time to linkage to care and viral suppression in the pre- versus post-implementation periods. Covariates included in the adjusted model were identified a *priori* and included age at HIV diagnosis, sex, race/ethnicity, HIV transmission risk factor, HIV testing (for the linkage to care outcome) or treatment (for the viral suppression outcome) site, and RWHAP enrollment status. We also conducted an inverse probability (IP)–weighted sensitivity analysis including all covariates in a propensity score model comparing post- to pre-implementation periods and fitting a marginal, IP-weighted model to avoid potential overfitting that may have been present in the multivariable model.

## RESULTS

We included 278 people newly diagnosed with HIV: 203 in the pre-implementation cohort and 75 in the post-implementation cohort. Overall, their median age was 30 years, 71% were male, 75% were Black non-Hispanic, 41% were MSM, and 67% were enrolled in the RWHAP. There were no differences in demographic characteristics, HIV testing site location, or HIV treatment site location in the pre- versus post-implementation periods. There was a decrease in the proportion of those reporting MSM transmission risk and an increase in the proportion with other/unknown HIV transmission risk factors in the post- versus pre-implementation periods. Conversely, there was an increase in the proportion of those enrolled in the RWHAP in the post- versus pre-implementation periods (Table 2).

**Table 2.** Demographic Characteristics of People Newly Diagnosed With HIV at Participating Connextion Sites in Memphis, Tennessee, January 2022–May 2025 (N = 278)

Characteristic	Pre-implementation (Jan 2022–May 2024) (n = 203)	Post-implementation (June 2024–May 2025) (n = 75)	*P* Value^a^
Age at HIV diagnosis, y, median (IQR)	30 (25–42)	29 (24–38)	.34
Sex, No. (%)			.72
Male	144 (71)	52 (69)	
Female	53 (26)	22 (29)	
Unknown	6 (3)	1 (1)	
Race/Ethnicity, No. (%)			.94
Black, non-Hispanic	153 (75)	56 (75)	
White, non-Hispanic	10 (5)	5 (7)	
Hispanic	34 (17)	12 (16)	
Other/Unknown	6 (3)	2 (3)	
HIV transmission risk factor, No. (%)			**.01**
MSM sexual contact	87 (43)	28 (37)	
Heterosexual contact	23 (11)	1 (1)	
IDU	2 (1)	1 (1)	
Other/Unknown	91 (44)	45 (60)	
HIV testing site			.06
A (CBO)	8 (4)	3 (4)	
B (FQHC)	73 (36)	21 (28)	
C (Health Department)	65 (32)	37 (49)	
D (Adult HIV Clinic)	57 (28)	14 (19)	
HIV treatment site			.76
B (FQHC)	95 (47)	36 (48)	
D (Adult HIV Clinic)	94 (46)	36 (48)	
E (Pediatric HIV Clinic)	14 (7)	3 (4)	
Baseline CD4^+^ count (cells/μL)			.14
<200	38 (19)	7 (9)	
200–499	79 (40)	35 (47)	
≥500	83 (42)	33 (44)	
Unknown	3 (0)	0 (0)	
Baseline HIV RNA (copies/mL)			.16
<200	25 (12)	12 (16)	
200–9999	38 (19)	18 (24)	
≥10 000	131 (65)	36 (48)	
Unknown	9 (4)	9 (12)	
RWHAP status			**.01**
Enrolled	127 (63)	60 (80)	
Not enrolled	76 (37)	15 (20)	

Abbreviations: CBO, community-based organization; FQHC, federally qualified health center; HIV, human immunodeficiency virus; IDU, injection drug use; IQR, interquartile range; MSM, male-to-male; RWHAP, Ryan White HIV/AIDS Program.

^a^Differences in categorical and continuous variables in the pre- versus post-implementation periods were tested using the Fisher exact and Wilcoxon rank-sum tests, respectively. Statistically significant findings are indicated with bold font.

Among 278 people newly diagnosed with HIV, the median time to linkage was 22 days pre-implementation and 18 days post-implementation (*P* = .18). Differences in median times to linkage by subgroup are displayed in Table 3. There were significantly lower median times to linkage among men (15 vs 26 days) and those receiving HIV testing at the health department (26 vs 47 days) in the post- versus pre-implementation periods, respectively.

**Table 3.** Median Time (Days) to Linkage to Care for People Newly Diagnosed With HIV at Participating Connextion Sites in Memphis, Tennessee, January 2022–May 2025 (N = 278)

Characteristic	Pre-implementation (Jan 2022–May 2024) (n = 203)	Post-implementation (June 2024–May 2025) (n = 75)	*P* Value^a^
Overall	22 (4–55)	18 (7–33)	.18
Age at HIV diagnosis, y			
<25	29 (15–69)	15 (4–37)	.05
25–34	26 (5–57)	15 (7–29)	.18
35–44	26 (8–60)	29 (11–33)	.63
45–54	13 (0–29)	15 (6–21)	.89
≥55	9 (4–21)	16 (11–38)	.13
Sex			
Male	26 (7–59)	15 (7–34)	**.04**
Female	11 (0–29)	18 (11–28)	.21
Unknown	35 (27–124)	36 (36–36)	.86
Race/Ethnicity			
Black, non-Hispanic	22 (7–55)	15 (7–33)	.22
White, non-Hispanic	15 (4–38)	29 (29–29)	.90
Hispanic	26 (0–57)	11 (0–23)	.14
Other/Unknown	13 (3–17)	49 (48–50)	.24
HIV transmission risk factor			
MSM contact	26 (11–55)	18 (7–34)	.12
Heterosexual contact	18 (5–35)	29 (29–29)	.56
IDU	152 (89–215)	29 (29–29)	1.00
Other/Unknown	15 (0–62)	15 (7–29)	.72
HIV testing site			
A (CBO)	29 (9–102)	7 (7–15)	.41
B (FQHC)	18 (7–36)	11 (7–26)	.31
C (Health Department)	47 (29–73)	26 (15–36)	**<.01**
D (Adult HIV Clinic)	4 (0–11)	5 (0–26)	.38
HIV treatment site			
B (FQHC)	26 (11–58)	24 (11–34)	.19
D (Adult HIV Clinic)	15 (0–47)	15 (4–29)	.67
E (Pediatric HIV Clinic)	31 (19–54)	36 (26–49)	1.00
Baseline CD4^+^ count (cells/μL)			
<200	13 (0–44)	15 (11–31)	.82
200–499	22 (7–62)	15 (7–31)	.30
≥500	22 (11–55)	18 (4–33)	.12
Baseline HIV RNA (copies/mL)			
<200	22 (7–55)	16 (6–31)	.41
20–9999	24 (11–62)	13 (5–32)	.13
≥10 000	18 (4–46)	20 (11–34)	.92
Unknown	47 (33–150)	11 (7–29)	.05
RWHAP status			
Enrolled	26 (7–58)	18 (7–34)	.16
Not enrolled	18 (4–47)	15 (9–33)	.70

Abbreviations: CBO, community-based organization; FQHC, federally qualified health center; HIV, human immunodeficiency virus; IDU, injection drug use; IQR, interquartile range; MSM, male-to-male; RWHAP, Ryan White HIV/AIDS Program.

^a^Differences in median times to linkage were tested using the Wilcoxon rank-sum test. Statistically significant findings are indicated with bold font.

Among 278 people newly diagnosed with HIV, the median time to viral suppression was 119 days pre-implementation and 62 days post-implementation (*P* < .01). Differences in median time to viral suppression by subgroup are displayed in Table 4. There were significantly lower median times to viral suppression among younger individuals (<25 years, 25–34 years, and 35–44 years). The decrease was also evident in males (128 vs 272 days); among Black individuals (69 vs 223 days); among MSM (84 vs 256 days); among individuals diagnosed at the FQHC (121 vs 194 days), health department (150 vs 274 days), and adult HIV (62 vs 219 days) sites; and among those receiving treatment at the FQHC (141 vs 234 days) and adult HIV (117 vs 281 days) sites in the post- versus pre-implementation periods. There was also a significant decrease in times among those enrolled (153 vs 245 days) and not enrolled (33 vs 194 days) in RWHAP.

**Table 4.** Median Time (Days) to Viral Suppression for People Newly Diagnosed With HIV at Participating Connextion Sites in Memphis, Tennessee, January 2022–May 2025 (N = 278)

Characteristic	Pre-implementation (Jan 2022–May 2024) (n = 203)	Post-implementation (June 2024–May 2025) (n = 75)	*P* Value^a^
Overall	223 (139–424)	137 (62–186)	<.01
Age at HIV diagnosis, y			
<25	216 (143–364)	137 (51–190)	**<.01**
25–34	267 (150–479)	154 (113–157)	**.02**
35–44	238 (150–391)	99 (64–172)	**.02**
45–54	219 (143–354)	110 (66–115)	.07
≥55	164 (117–289)	159 (148–171)	.96
Sex			
Male	272 (169–461)	128 (80–179)	**<.01**
Female	154 (113–245)	157 (127–187)	.52
Unknown	464 (263–537)	153 (153–153)	1.00
Race/Ethnicity			
Black, non-Hispanic	223 (135–424)	69 (36–113)	**<.01**
White, non-Hispanic	312 (249–411)	186 (124–194)	.13
Hispanic	194 (113–316)	146 (51–157)	.08
Other/Unknown	440 (191–696)	110 (110–110)	.40
HIV transmission risk factor			
MSM contact	256 (153–418)	84 (58–153)	**<.01**
Heterosexual contact	223 (150–512)	NA	NA
IDU	280 (206–354)	NA	NA
Other/Unknown	216 (121–393)	154 (117–194)	**.01**
HIV testing site			
A (CBO)	256 (232–303)	NA	NA
B (FQHC)	194 (119–390)	121 (66–170)	**<.01**
C (Health Department)	274 (144–482)	150 (114–199)	**.03**
D (Adult HIV Clinic)	219 (144–356)	62 (51–157)	**<.01**
HIV treatment site			
B (FQHC)	234 (137–393)	141 (81–197)	**<.01**
D (Adult HIV Clinic)	281 (150–512)	117 (57–157)	**<.01**
E (Pediatric HIV Clinic)	143 (113–202)	146 (146–146)	1.00
Baseline CD4^+^ count (cells/μL)			
<200	219 (136–482)	117 (99–122)	.08
200–499	254 (170–465)	133 (59–160)	**<.01**
≥500	205 (128–406)	157 (110–201)	**.04**
Baseline HIV RNA (copies/mL)			
<200	194 (102–292)	102 (32–154)	**.02**
200–9999	216 (148–398)	153 (88–208)	.09
≥10 000	254 (147–447)	146 (113–192)	**<.01**
RWHAP status			
Enrolled	245 (153–453)	153 (112–190)	**<.01**
Not enrolled	194 (106–362)	33 (24–53)	**<.01**

Abbreviations: CBO, community-based organization; FQHC, federally qualified health center; HIV, human immunodeficiency virus; IDU, injection drug use; IQR, interquartile range; MSM, male-to-male; NA, not applicable (no individuals contributing data within that demographic category); RWHAP, Ryan White HIV/AIDS Program.

^a^Differences in median time to linkage were tested using the Wilcoxon rank-sum test. Statistically significant findings are indicated with bold font.

**Figure 1.**
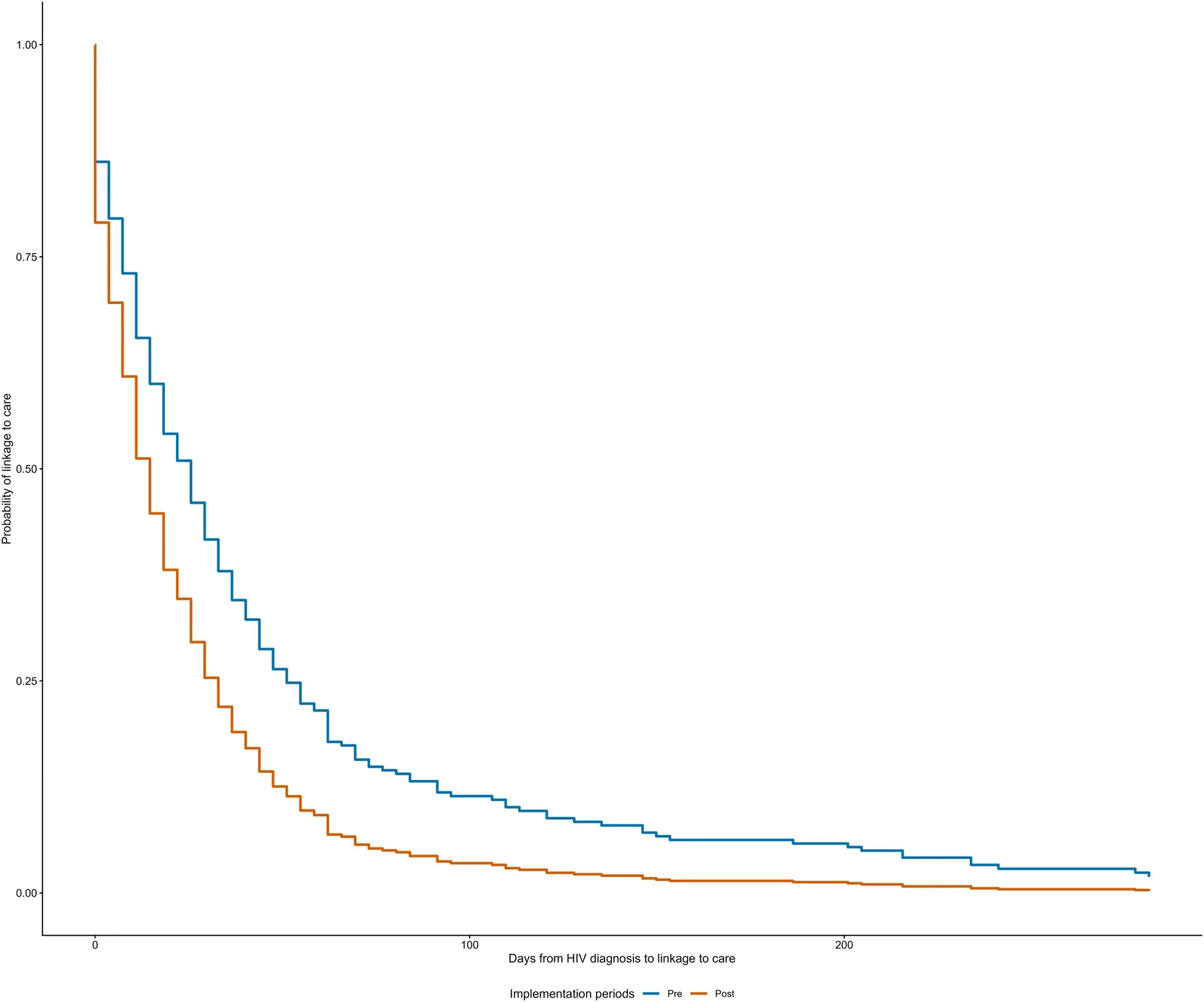
Adjusted survival curves for time to linkage to care stratified by pre- and post-implementation periods among people newly diagnosed with human immunodeficiency virus (HIV) at participating Connextion sites in Memphis, Tennessee, January 2022–May 2025. Curves are estimated at the means of covariates included in the multivariable Cox regression model. Individuals with >12 months between HIV diagnosis and first laboratory measure were censored at 12 months for purposes of time-to-event comparisons. The number of individuals at risk reflects those who are still under observation and have not yet been linked to care or censored as of the time indicated on the x-axis. The number of events reflects the number of individuals who have been linked to care as of the time indicated on the x-axis. Covariates included in the adjusted model were identified a priori and included age at HIV diagnosis, sex, race/ethnicity, HIV transmission risk factor, HIV testing (for the linkage to care outcome) or treatment (for the viral suppression outcome) site, and Ryan White HIV/AIDS Program enrollment status.

There was a significant increase in the adjusted probability of linkage to care (aHR, 1.76 [95% CI, 1.31–2.36]; Figure 1) in the post-implementation period compared to the pre-implementation period. In the IP-weighted sensitivity analysis, the marginal probability of linkage to care remained substantially higher in the post- versus pre-implementation period (aHR, 1.90 [95% CI, 1.50–2.40]) (Supplementary Table 1).

**Figure 2.**
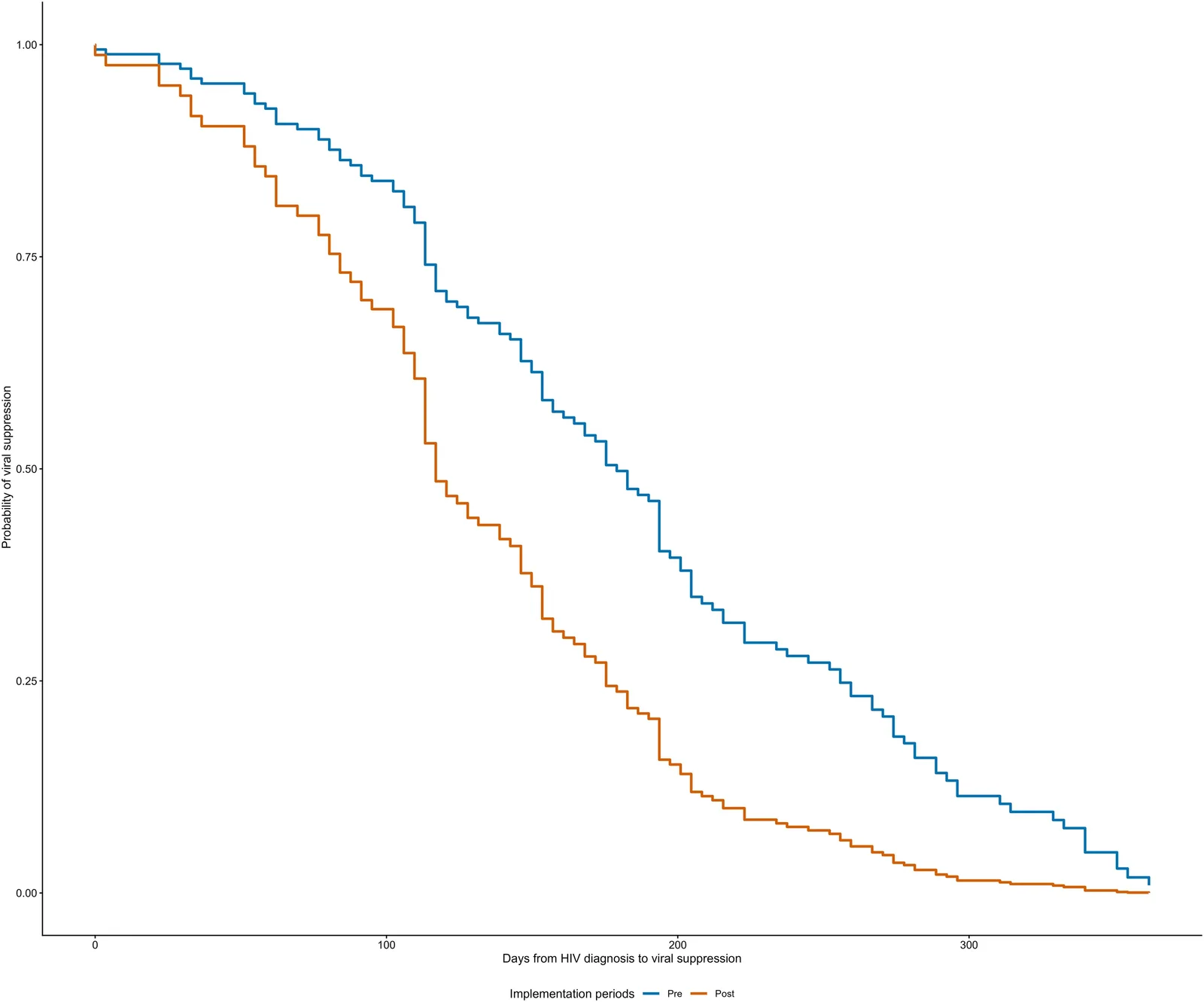
Adjusted survival curves for time to viral suppression stratified by pre- and post-implementation periods among people newly diagnosed with human immunodeficiency virus (HIV) at participating Connextion sites in Memphis, Tennessee, January 2022–May 2025. Curves are estimated at the means of covariates included in the multivariable Cox regression model. Individuals with >12 months between HIV diagnosis and first laboratory measure were censored at 12 months for purposes of time-to-event comparisons. The number of individuals at risk reflects those who are still under observation and have not yet achieved viral suppression or been censored as of the time indicated on the x-axis. The number of events reflects the number of individuals who have achieved viral suppression as of the time indicated on the x-axis. Covariates included in the adjusted model were identified a priori and included age at HIV diagnosis, sex, race/ethnicity, HIV transmission risk factor, HIV testing (for the linkage to care outcome) or treatment (for the viral suppression outcome) site, and Ryan White HIV/AIDS Program enrollment status.

There was also a significant increase in the adjusted probability of viral suppression (aHR, 4.22 [95% CI, 2.67–6.69]; Figure 2) in the post-implementation compared to the pre-implementation periods. In the IP-weighted sensitivity analysis, the marginal probability of viral suppression within 12 months of HIV diagnosis remained substantially higher in the post- versus pre-implementation period (aHR, 4.09 [95% CI, 2.89–5.78]) (Supplementary Table 1).

## DISCUSSION

The implementation of the Connextion (English) or Connextión (Spanish) rapid ART intervention among participating sites in Memphis, Tennessee, resulted in a significant decrease in the times to both linkage to care and viral suppression for people newly diagnosed with HIV. This was a multicomponent intervention with changes to the processes for HIV testing, linkage to care handoffs from testing to treatment facilities, and use of pharmaceutical samples for ART initiation. Importantly, the Connextion intervention was developed using implementation science methods to ensure local relevance, and the intervention could be further adapted by the participating sites to address their specific needs as they arise in the future [7, 20].

Other demonstration projects across the US South have identified similar findings, although there have been variations in the rapid ART implementation strategies. For example, a rapid ART initiative for people newly diagnosed with HIV in New Orleans, Louisiana (a Medicaid expansion state) took place at an FQHC. To increase provider availability for rapid-start visits, the program used a non-HIV specialist at the first visit and transitioned to an HIV specialist for subsequent care [10]. This approach was taken by the FQHC site participating in Connextion but was not an option for the two dedicated HIV clinics. This strategy, referred to as task shifting, involves delegating tasks from more specialized to less specialized healthcare workers to provide more specialized services where there are workforce shortages [21] and has been recommended by the World Health Organization for HIV care [22].

Another rapid ART initiative in Jackson, Mississippi (a Medicaid nonexpansion state) took place at a CBO that leveraged pharmaceutical samples and medication assistance programs [13]. Pharmaceutical samples were already being utilized by the two dedicated HIV clinics prior to Connextion implementation. However, the FQHC site more recently implemented providing pharmaceutical samples in collaboration with their on-site pharmacies after Connextion implementation. As part of this work, local site internal policies for the storage and distribution of pharmaceutical samples were developed specifically for HIV medications but were later expanded to include non-HIV medications benefiting all FQHC clients, regardless of HIV status.

Two other large rapid ART initiatives implemented in the southern US took place at dedicated HIV clinics associated with academic, safety-net hospitals in Medicaid nonexpansion states, like Tennessee. In Atlanta, Georgia, implementation strategies included removal of administrative barriers to rapid ART initiation, including proof of residence/income and addition of open provider slots dedicated to rapid ART initiation [11]. In Miami, Florida, implementation strategies included waiving the need for documentation of a positive confirmatory HIV test result and assistance from the Florida Department of Health in funding the first 30 days of HIV care [12]. Memphis–Shelby County had already adopted presumptive RWHAP eligibility with a 30-day window to provide proof of residence/income and documentation of positive confirmatory HIV test result prior to the Connextion rapid ART intervention pre-implementation period [23]. However, rapid confirmatory HIV testing was implemented as part of the Connextion rapid ART intervention at the health department testing site [24]. These differences highlight the need to use systematic implementation science methods to identify rapid ART strategies best suited to each locality's context.

This study has several limitations. First, this is a non-randomized, observational study, so there may be other events occurring over the study period that contributed to improved outcomes aside from the Connextion implementation. Importantly, the first year of the pre-implementation period occurred during the COVID-19 pandemic, which may have led to transient disruptions in HIV care. Second, was our use of CD4^+^ and HIV RNA laboratory values as a proxy for a care visit, although we did not have access to HIV visit dates via public health surveillance data. Relatedly, the frequency of CD4^+^ and HIV RNA laboratory tests was not conducted systematically at regular intervals, but rather as deemed necessary by the HIV care providers. However, we expect this limitation to bias our results toward the null because if laboratory tests were conducted systematically at regular intervals, we would identify linkage to care and viral suppression sooner than if we wait for the laboratory tests to be conducted as part of clinical care. Third, this was a study conducted in a single southern US city, so results may not be generalizable to other settings although the implementation science methods used in the strategy development could be applied in other high-HIV-burden areas seeking to develop local rapid ART models [7]. Fourth, follow-up is currently limited to 1 year post-implementation, so it is unclear if these results will persist over longer periods of follow-up. Continued outcomes assessment over longer-term follow-up periods will be important to assess Connextion intervention sustainability.

Last, we are unable to determine which components of the Connextion implementation were most impactful on linkage and viral suppression outcomes. We did not have access to data on clinic visit dates or ART initiation dates, so linkage to care outcomes were based on the first available CD4^+^ or HIV-1 RNA value in public health surveillance data. Our findings of a stronger effect on viral suppression than on linkage to care may be related to a stronger impact of the Connextion implementation on decreasing the time from the first HIV treatment visit to ART initiation compared to the time from HIV diagnosis to the first HIV treatment visit. In fact, almost half of the people newly diagnosed with HIV were linked to HIV care at the FQHC site that only began to provide same-day ART initiation in the post-implementation period. Qualitative interviews are currently being conducted among HIV testing and treatment site staff and people newly diagnosed with HIV to assess implementation outcomes, including fidelity to the individual Connextion components, and to elucidate which components of the intervention were most impactful.

The Connextion intervention evaluation has several important strengths. This intervention was successful in a region of the country and EHE jurisdiction with high HIV incidence [16], in a state without Medicaid expansion [17], and in an MSA with a high poverty rate [18]. Importantly, after implementation of Connextion there was an approximately 50% decrease in the time to viral suppression among people newly diagnosed with HIV including among young, Black MSM, a population disproportionately impacted by HIV nationally [16]. The intervention resulted in a published SOP and formal execution of MOUs across a variety of types of clinics including a CBO, FQHC, municipal health department, and academically affiliated HIV clinics.

In conclusion, we developed a model that was successful in decreasing the time to linkage to care and viral suppression with the use of implementation science to identify optimal rapid ART implementation strategies for Memphis overall and for each participating site. Current efforts are focused on ensuring the sustainability of the rapid ART model by transitioning the oversight of the intervention from the study team to local partners. Future efforts will include recruitment of additional HIV testing sites and treatment sites in Memphis to participate in this intervention and expansion of eligible participants from people newly diagnosed with HIV to those living with HIV who have been out of care.

## Supplementary Material

ofag506_Supplementary_Data

## Supplementary Data

Supplementary materials are available at *Open Forum Infectious Diseases* online. Consisting of data provided by the authors to benefit the reader, the posted materials are not copyedited and are the sole responsibility of the authors, so questions or comments should be addressed to the corresponding author.
