# Supplementary material for: Transforming Human Immunodeficiency Virus Outcomes: Impact of a Rapid Antiretroviral Therapy Model on Linkage to Care and Viral Suppression in Memphis, Tennessee
Source: Open Forum Infect Dis. 2026 Aug 19;13(9):ofag506. doi: 10.1093/ofid/ofag506 (PMC13542720; doi:10.1093/ofid/ofag506)
Supplement: Supplementary file 1 [file ofag506_supplementary_data.docx]

**Supplemental Table 1**: Results from inverse probability (IP)-weighted marginal Cox regression using a propensity score model for membership in the pre- vs. post-intervention period

| Outcome for Marginal Cox Model | | Marginal (IP-weighted) coefficient for post- vs. pre-intervention period | | 95% Confidence Interval | |
| --- | --- | --- | --- | --- | --- |
| Linkage to Care | **1.90** | | 1.50 – 2.40 | |  |
| Viral Suppression | **4.09** | | 2.89 – 5.78 | |  |

**Bold font** indicates coefficients which differ from the null with p<0.05
